# Early life opportunities for prevention of diabetes in low and middle income countries

**DOI:** 10.1186/1471-2458-12-1025

**Published:** 2012-11-23

**Authors:** Mark A Hanson, Peter D Gluckman, Ronald CW Ma, Priya Matzen, Regien G Biesma

**Affiliations:** 1 Institute of Developmental Sciences, Faculty of Medicine, University of Southampton, Mailpoint 887, Southampton General Hospital, Tremona Road, Southampton, SO16 6YD, UK; 2 Liggins Institute, University of Auckland, Auckland, Private Bag 92019, Auckland, 1023, New Zealand; 3 Singapore Institute of Clinical Sciences, Brenner Centre for Molecular Medicine, 30 Medical Drive, Singapore, 117609, Singapore; 4 Department of Medicine and Therapeutics, Chinese University of Hong Kong, Shatin, Hong Kong, SAR, China; 5 Hong Kong Institute of Diabetes and Obesity, Chinese University of Hong Kong, Prince of Wales Hospital, Shatin, Hong Kong, SAR, China; 6 Novo Nordisk A/S, Novo Allé, DK-2880, Bagsværd, Denmark; 7 Department of Epidemiology and Public Health Medicine, Royal College of Surgeons in Ireland, 123 St Stephens Green, Dublin 2, Ireland

**Keywords:** Adolescents, Diabetes, Health literacy, Interventions, Life-course, Non-communicable diseases, Gestational diabetes mellitus, Obesity

## Abstract

**Background:**

The global burden of diabetes and other non-communicable diseases is rising dramatically worldwide and is causing a double poor health burden in low- and middle-income countries. Early life influences play an important part in this scenario because maternal lifestyle and conditions such as gestational diabetes and obesity affect the risk of diabetes in the next generation. This indicates important periods during the lifecourse when interventions could have powerful affects in reducing incidence of non-communicable diseases. However, interventions to promote diet and lifestyle in prospective parents before conception have not received sufficient attention, especially in low- and middle-income countries undergoing socio-economic transition.

**Discussion:**

Interventions to produce weight loss in adults or to reduce weight gain in pregnancy have had limited success and might be too late to produce the largest effects on the health of the child and his/her later risk of non-communicable diseases. A very important factor in the prevention of the developmental component of diabetes risk is the physiological state in which the parents enter pregnancy. We argue that the most promising strategy to improve prospective parents’ body composition and lifestyle is the promotion of health literacy in adolescents. Multiple but integrated forms of community-based interventions that focus on nutrition, physical activity, family planning, breastfeeding and infant feeding practices are needed. They need to address the wider social economic context in which adolescents live and to be linked with existing public health programmes in sexual and reproductive health and maternal and child health initiatives.

**Summary:**

Interventions aimed at ensuring a healthy body composition, diet and lifestyle before pregnancy offer a most effective solution in many settings, especially in low- and middle-income countries undergoing socio-economic transition. Preparing a mother, her partner and her future child for “the 1000 days”, whether from planned or unplanned conception would break the cycle of risk and demonstrate benefit in the shortest possible time. Such interventions will be particularly important in adolescents and young women in disadvantaged groups and can improve the physiological status of the fetus as well as reduce the prevalence of pregnancy conditions such as gestational diabetes mellitus which both predispose to non-communicables diseases in both the mother and her child. Pre-conception interventions require equipping prospective parents with the necessary knowledge and skills to make healthy lifestyle choices for themselves and their children. Addressing the promotion of such health literacy in parents-to-be in low- and middle-income countries requires a wider social perspective. It requires a range of multisectoral agencies to work together and could be linked to the issues of women’s empowerment, to reproductive health, to communicable disease prevention and to the Millennium Development Goals 4 and 5.

## Background

The rising incidence of diabetes in both developed and developing countries [1-3] is generating a large humanitarian and financial burden [4]. For example, at least 93 million people are affected by diabetes in China alone [5]. Especially worrying is the generalized fall in the age at onset of diabetes [2,6]. With rapidly increasing globalization, lifestyles in low- and middle-income countries (LMICs) increasingly include high fat and calorie-dense diets and inadequate physical exercise and are resulting in an increased worldwide burden of Non-Communicable Diseases (NCDs) [7]. LMICs thus now face a double burden - continued high rates of infectious diseases and rapidly growing NCD prevalence. Globally, 85% of all people with undiagnosed diabetes are in LMICs, where resources are often lacking and governments may not prioritize screening for the condition [3]. In addition, the interaction between diabetes and major infectious diseases has a large impact on public health within developing countries (such as in Sub-Saharan Africa) and adversely affects progress towards the Millennium Development Goals (MDGs) [8-10]. Poverty, inequality, lack of education and the nature of the nutritional transition are root causes of the problem [11] while limited resources mean that NCDs must compete for political attention and financial investment [10]. Following several recent regional and international meetings and initiatives [12] including the UN high-level meeting on prevention and treatment of NCDs from 19-20 September 2011 [13], the global threat of diabetes and other NCDs is beginning to receive greater political attention. Until recently, there was much publicity for the view that diabetes and NCDs were largely manifest in adults as a result of the biblical sins of ‘gluttony and sloth’, so there was both an urgent need to get individuals to take more responsibility for their adult lifestyle and to discourage the food industry from producing the ‘unhealthy’ foods which fuelled this epidemic of disease [8]. However, a substantial body of evidence makes it clear that early life influences also play an important part here, and that maternal (and, to a degree, paternal) lifestyle and conditions such as gestational diabetes affect not only prenatal and infant development but also the adequacy of responses to later challenges such as an obesogenic lifestyle. This emphasises the importance of NCD risk reduction starting in adolescents and young women before conception and during pregnancy [14].

In this review, we examine the role of early life influences on diabetes risk, and argue that interventions to promote diet and lifestyle in prospective parents before conception have not received sufficient attention, especially in LMICs undergoing socio-economic transition. Such interventions will be particularly important in adolescents and young women and seem likely not only to reduce the prevalence of NCDs in the next generation but also to reduce that of pregnancy conditions such as gestational diabetes mellitus (GDM), which predispose to NCDs in both the mother and her child. We therefore propose that greater emphasis on promotion of health literacy, coupled with screening for risk and management of chronic health conditions such as obesity and diabetes, prior to conception will be an important additional approach to tackling the epidemic of NCDs. If effective, this may be a cost-effective way of addressing the epidemic of NCDs such as diabetes.

## Discussion

### Early life influences on diabetes risk

We have argued that diabetes incidence is rising so fast in young people in LMICs not solely because of the adoption of attributes of Western lifestyle, but also because the evolved biology of human early life has not prepared individuals for such a challenge – the mismatch hypothesis [14]. The operation of maternal constraint [15,16], by which processes operate to match the growth of the fetus to maternal body proportions such as pelvic dimensions, occurs in all pregnancies, and thus makes each of us potentially mismatched to our postnatal environment. However, the degree of this mismatch varies. The processes of maternal constraint, and thus the risk of later mismatch, appear to operate more powerfully in teenage pregnancies and in primipara. Thus interventions targeted to this time in the mother’s lifecourse may be particularly effective in reducing NCD risk in the next generation.

**Figure 1:**
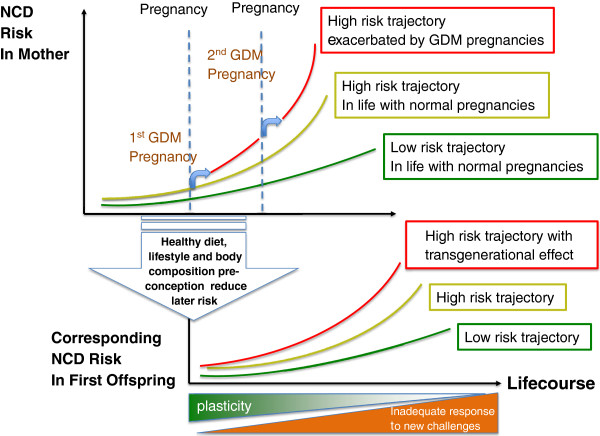
The risk of NCD in mother and offspring increases with each pregnancy.

A body of knowledge is accumulating which suggests that environmental factors act in early life via epigenetic factors to set the development of many aspects of the offspring phenotype, contributing to potential later mismatch [17]. This model (see Figure 1) posits that critical components of risk are established early in development, when information about the current environment affects the settings of homeostatic control processes influencing the individual’s responses to their later environment: the underlying mechanisms involve the evolutionary conserved processes of developmental plasticity [17,18]. Central to developmental plasticity is the concept that the mother is able to pass on information about the external environment during pregnancy and nursing of her infant. The level and fidelity of these processes is influenced by a wide range of factors including mother’s genetic makeup, her early life, her current nutritional status, age, body composition and proportions, lifestyle (especially level of physical activity, smoking, alcohol consumption), parity and external stressors such as infection, pollution etc. [19]. Hence aspects of maternal phenotype and trans-generational environmental changes can lead to increased risk of disease in the offspring when what has been promised about the environment, ‘inferred’ from intrauterine or neonatal cues, turns out not to be accurate. Such a mismatch between the biology induced during prenatal life and later environment has been suggested to increase the risk of NCDs being passed from one generation to the next; it is supported by extensive experimental and clinical data [17,20].

The risk of NCD such as diabetes is present in everyone and increases throughout the lifecourse. In women the insulin resistance of pregnancy increases such risk (upward excursions shown here for two pregnancies), particularly of GDM but also of type 2 diabetes and cardiovascular disease. This contributes to transmission of NCD risk to the next generation. Early development affects the trajectory of this risk, thus a focus on health before conception reduces NCD risk in both the mother and her children (based on Godfrey et al., 2010 [19] and Sattar and Greer, 2002 [43].

### Diabetes begetting diabetes and the role of gestational diabetes and maternal obesity

The processes noted above do not constitute the only developmental pathway leading to intergenerational passage of risk of NCDs. It is alarming that an increasing number of women develop type 2 diabetes during their reproductive years [21,22]. Epidemiological studies show that children born to type 1 or 2 diabetic mothers also have a greater susceptibility to diabetes and obesity in later life [23,24]. That this risk is related to intra-uterine exposure to hyperglycaemia is shown by the observation that, among siblings, the risk of diabetes is higher in those born after the mother was diagnosed with diabetes [25]. These observations have been extended recently, as offspring exposed even to mild hyperglycaemia during pregnancy have increased adiposity and are at increased risk of later diabetes and cardiometabolic disease [26,27]. Through perpetuation of the cycle of ‘diabetes begetting diabetes’, these factors are driving further escalation of the epidemic of NCDs [28,29]. Recent animal data now also raise the possibility of paternal transmission of diabetes risk between generations [30] so the focus of potential interventions should not only be on women.

The rising prevalence of maternal obesity is of particular concern in populations undergoing rapid socio-economic transitions [1,2,21]. Obesity is associated with an increased risk of many pregnancy complications such as hypertension, preeclampsia, and GDM. Evidence shows that a child of an obese mother may suffer from exposure to a suboptimal environment *in utero* and is also more likely to become obese [31,32]. Experimentally feeding pregnant animals a high fat diet gives rise to offspring who become overweight and who demonstrate a range of metabolic and functional disorders similar to the human metabolic syndrome and which have now been shown to be associated with epigenetic changes such as DNA methylation at components of genes involved in hepatocyte production and metabolism, [33]. Pregnancy represents a state of relative maternal insulin resistance, which helps promote the transfer of nutrients such as glucose, fatty acids and amino acids to the fetus [34]. Placental nutrient transfer is determined by the concentration gradient, blood flow and the operation of active and facilitated transporters [35]. However, in contrast to amino acids, there is no upper limit to placental transfer of glucose as maternal blood levels rise, suggesting that the rates of glucose exposure of the fetus that are often now experienced are novel in evolutionary terms. As a result, moderately increased fetal glucose supply will lead to fetal hyperinsulinaemia and a small increase in lean body as well as fat mass [36-39]. This may be viewed as adaptive as in the neonatal period relative adiposity provides metabolic reserves for thermogenesis and critical organ function in the event of inadequate maternal care [18]. GDM can be envisaged as the more extreme outcome of physiological processes, when maternal insulin resistance is accentuated by the woman’s own developmental, genetic and environmental circumstances: for example, women of lower birth weight have a greater risk of developing GDM [37], whilst genetic variants associated with type 2 diabetes are also associated with increased risk of GDM [40,41]. In the evolutionary mismatched situation of over-nutrition and low levels of physical activity, now increasingly common for women in their reproductive years, such mechanisms contribute not only to the rise in GDM but to that in obesity and diabetes in their children, and play an important role in perpetuating a vicious cycle of disease. For example, a recent paper shows that in Canadian first nation peoples up to 30% of the incidence of type 2 diabetes has its origin in GDM in the previous generation [42]. Conditions such as GDM carry risk of cardiovascular disease for both the mother as well as the child, a risk which increases with each pregnancy (Figure 1) [43]. These findings have significant long-term implications for global public health. Now more than ever, effective strategies for preventing GDM are needed.

### Current public health strategies in adults have limited success

Most interventions to promote diet and lifestyle aimed at reducing overweight/ obesity, and thus the risk of NCDs such as diabetes later in life, have focused on adults. However, evidence suggests that interventions aimed at weight loss in adults or to prevent excessive weight gain during pregnancy and subsequent weight retention have had limited success. First, weight loss and persistent changes in lifestyle are difficult to sustain in adults because the neuro-endocrine changes that drive appetite remain unaltered [44]. There is growing evidence of antenatal and infant determinants of appetite and food preference that persist through adult life [45-47]. Therefore relying on adult reversal of weight gain as a preventative approach is a flawed strategy in developed countries and is even more likely to be flawed in less developed societies. In addition, greater maternal pre-pregnancy weight and gestational weight gain up to 36 weeks of gestation are associated with greater offspring adiposity and adverse cardiovascular risk factors [48]. Finally, recent evidence shows that risk factors for diabetes and cardiovascular disease are no greater in non-obese adults who had been obese children than they are in individuals who had never been obese, but are clearly less than those in people who became obese as adults or who were obese both as children and adults [49]. This makes a strong case for focusing earlier in life before appetite control, food preference and fat cell number are established, and before obese children reach adulthood.

### Intervening from conception – the 1000 days

There has been much publicity on the 1000 days campaign to improve nutrition of mothers and children in developing countries launched by the 1^st^ Lady of the USA ( http://www.thousanddays.org/). This period of life covers a woman’s pregnancy and the first 2 years of her child’s postnatal life, a time when the processes of developmental plasticity operate powerfully to influence offspring phenotype and when excessive growth, as determined by crossing of more than 2 centiles is associated with later adiposity in childhood [50]. Despite a unique window of opportunity to shape the healthy future of mothers and children during this period, findings are inconsistent in relation to which factors need to be targeted in interventions to reduce the risk of later NCDs such as diabetes. First, even though evidence shows that preventing excessive gestational weight gain improves maternal, neonatal and pregnancy outcomes such as the risk of preeclampsia, preterm delivery and gestational diabetes [51-54], there is limited evidence of interventions to manage weight during this period being effective [55,56]. Poor intervention effectiveness seems to be related to inadequately addressing (social) barriers that women experience in achieving a healthy weight gain in pregnancy, such as a perceived lack of control and contradictory and conflicting messages about nurturing behaviours during pregnancy [56]. Pregnancy is a time of when many influences operate on parents and it may not be the best time for messages to be delivered about preventing excessive weight gain in women. Secondly, there is a growing body of evidence that breast feeding for at least 4 months leads to a reduction in long-term obesity in the child [57,58] and that the introduction of solids before the age of 4 months both in formula- or breast-fed infants is associated with later obesity [59]. A recent study [60] suggests that self-weaned children are less likely to become obese, showing that relatively subtle changes in infant care may have long-term effects. Despite the overwhelming evidence that breastfeeding benefits mother and child, current breastfeeding rates worldwide are far from optimal, particularly among low-income, overweight and obese women [61,62]. Breastfeeding initiation and duration, and its exclusivity, are positively associated with the age, educational level and SES of the mother, while there is an inverse pattern for early formula and cow’s milk consumption [62]. Again, a woman's ability to adhere to health recommendations (in this case to infant feeding practices) seems to be influenced by a wide range of socio-economic and cultural variables. In contrast to intervention to reduce excessive weight gain during pregnancy, treatment of mild gestational diabetes was associated with reduced risk of fetal overgrowth or adiposity at birth [63,64].

There is some evidence that home-based early mentoring interventions, focusing on interpreting infants' cues, non-food methods of managing infant behavior, and mother-grandmother interactions are effective in improving infant feeding practices and delaying early complementary infant feeding among adolescent and low-income mothers [65,66]. This indicates that multiple types of interventions, including community based strategies such as educating young girls, the wider family and the social network, are needed to address this complex health problem, especially in disadvantaged groups.

### Potential early life interventions- intervening before conception is critical

A very important factor in the prevention of the developmental component of diabetes risk is the physiological state in which the parents enter pregnancy – i.e. the diet, body composition and lifestyle of the mother and to a lesser extent the father. There is considerable data from experimental studies in a range of animal species and prospective studies in humans that the mother’s diet and body composition before and in early pregnancy are related to phenotypic characteristics of the child, such as adiposity at birth and in childhood, and markers of cardiovascular risk such as carotid artery intima-media thickness [67-69]. The processes involved in influencing embryonic and fetal development are gradually being unravelled. Both animal and human studies reveal effects on placental function [70], the development of fetal organ function and on epigenetic processes in the offspring early in development [71]. Most couples will not be aware that they have conceived until after about 6-8 weeks of pregnancy, especially in societies where a high proportion of pregnancies are unplanned. Lower SES women tend to link with health care professionals later in pregnancy. Thus interventions in mid-late pregnancy will be too late to produce the largest effects on the health of the child and his/her later risk of NCDs. Moreover teenage pregnancy occurs at a time when the girl is still growing: resources are allocated to this growth rather than to her fetus, further compromising its development. In recently conducted research in the UK, it is clear that maternal diabetes during pregnancy is accompanied by a greater risk of birth defects and complicated pregnancy outcomes [41]. Therefore it is important that women and their partners are screened for risk factors before they plan pregnancy to improve health outcomes for both the mother and offspring.

The relation between overweight/ obesity and NCD such as diabetes, is clearly not simple [72] and interventions need to be multifaceted and incorporated into social context. So far, very few interventions to reduce childhood obesity have been set up at the community level [73] and outside the school environment [74]. Community-based interventions are particularly important in societies where teenage girls who may be married at a very young age and become pregnant soon after, or who may have an unplanned pregnancy, and are more likely to cease attending school. We argue that focussing interventions on the adolescent girl, and her partner, necessitates promotion of health literacy, such as lifestyle, family planning and the importance of breastfeeding. Health literacy means more than being able to read pamphlets and successfully make appointments. By improving people’s access to health information and their capacity to use it effectively, health literacy is critical to empowerment [75]. Health literacy in adolescents is more likely to be taken to the highest level of critical thinking about health and lifestyle if it is made context-specific and conducted outside the school environment, which is associated with authority and which may not be available to many girls in particular. Low health literacy affects the cognitive and social skills that determine the motivation and ability of women to gain access to, understand, and use information to engage in health promotion and prevention activities both for her and her (future) children [76-78]. Children of parents with higher literacy skills are more likely to have better outcomes in child health promotion and disease prevention [79]. It has been suggested that health education interventions that include clearly written education material that are appropriate for (future) parents’ health knowledge with brief counselling would be an effective strategy to reduce health literacy-related disparities [76,77].

### Delivering pre-conception interventions in LMICS

Delivering these interventions should be done carefully and after identifying the right channels. In many countries the existing public health programmes which focus on sexual and reproductive health may offer opportunities to deliver such interventions in a cost effective manner to adolescents and women of reproductive age. However, our experience [80] is that effective programmes may need to pay close attention to the pedagogical approach used which will have to be tailored to the community. It is important that measures of harm or unintended consequences are included in evaluations targeting eating and activity related behaviours in adolescents, where body image sensitivities are common and could possibly result in causing unintended consequences such as stigmatisation, low self-esteem or unhealthy dieting practices [81]. An additional challenge in trying to improve adolescents’ nutrition or physical activity is that some cultures have less negative views on overweight individuals and a larger body type is accepted socio-culturally, or even found desirable as a sign of prosperity or being HIV negative. It will be critical to incorporate the positive elements of such culture regarding body image and food rather than attempting to shift values toward those of Western culture [82].

Four million neonatal deaths occur every year [83], many in LMICs, and there is universal concern that the goal of MDG-4 to promote child survival will not be met in most settings. Considerable reductions in this mortality, in a cost-effective manner, can be achieved by implementing an integrated programme combining outreach and family-community care with facility-based clinical services [84]. In our view it is important that emphasis on preventing maternal and neonatal mortality does not occur at the expense of adopting a longer-term lifecourse strategy, starting pre-conception [85], needed for NCD prevention. However it is pleasing to note that the Political Declaration at the High Level meeting on NCDs at the UN in Sept 2011 specifically recognised the important role of maternal and child health in NCD prevention (clause 26) [13].

Based on the evidence presented in this review, we believe that real opportunities exist for developing community-based family-lifestyle change interventions to improve access to and use of reliable information on NCDs and to promote a healthy lifestyle among adolescents and young women in LMICs. There is an urgent need to conduct research on pilot schemes tailored to this subgroup. These will need to be based in the community and relate to socio-economic factors [84,85], be culturally appropriate and context specific and involve primary health care workers, schools, parents and caregivers and other social organisations. They may need to include simple health-monitoring or exercise tests and screening for blood glucose, for which the results can be used to monitor effectiveness and also fed back to participants to maintain motivation and self-efficacy [81]. Such programmes are perhaps best piloted in societies where there is a proven track-record in health promotion, e.g. through HIV-AIDS programmes, and where there is existing infrastructure to accelerate results. Indeed, it will be important that such pilot schemes are integrated into, rather than compete with, other programmes in sexual health, drug, smoking and alcohol consumption reduction etc. Such a programme would then dovetail with antenatal health monitoring programmes, and with the early diagnosis and prevention of conditions such as GDM. For the latter, many of the first line intervention options such as diet, physical exercise and weight control are similar to the behaviours to be promoted in adolescents before conception. The programme would similarly link effectively with MCH initiatives aimed at reducing preterm- and stillbirth and maternal mortality and with postnatal healthcare programmes, aimed at promoting breast feeding, healthy supplementary and weaning foods, vaccination etc. The burden of NCDs impacts heavily on women’s health and integrating NCD prevention and control into maternal and child health programmes will improve access to NCD services [86]. This approach requires an agenda broader than health agencies alone and it would be desirable that a broader range of global agencies such as UNDP, UNESCO, UNICEF, and UN Women became active alongside WHO [87].

## Discussion: seeing the bigger picture

The long-term consequences of a poor start to life include greater risk of NCDs such as diabetes and cardiovascular disease as well as greater risks of childhood morbidity and mortality and impaired cognitive and emotional development. Just as cycles of disadvantage in one generation lead to disadvantage in the next, obesity, gestational diabetes, maternal ill health and under-nutrition can lead to health disparities and disease in the next generation. The perceived long time lag between instituting an intervention in early life and detecting a beneficial effect on NCD incidence in adults has inevitably reduced the sense of urgency in implementing such interventions, particularly in the context of short-term outcomes such as maternal or child mortality and communicable disease. However we are now in a position to use shorter-term intermediate outcome markers of later disease risk to monitor and promote a healthy start to life programmes such as those in pre-conception care. Such programmes might demonstrate: a reduction in cardio-metabolic risk factors in adolescents; reduced occurrence or severity of GDM; improvement in pregnancy outcomes such as macrosomia, dystocia and hypoglycaemia of the neonate. For such interventions the evidence base is strong. For other intermediate outcome markers, such as a healthier profile of epigenetic markers at birth in umbilical cord, placenta or cord blood, the evidence base is rapidly accumulating. Postnatally there are also opportunities to use outcome markers, for example trajectories of growth in the first two years [50], profiles of inflammatory and other biomarkers in children [88]; and incidence of some infant and early childhood disorders such as wheeze and asthma [89]. Clearly, the earlier and intervention is instituted, the more likely it is to be effective. There is thus an urgent need to conduct pre-conception interventions, to measure their efficacy and conduct cost-benefit analysis of this approach to NCD risk reduction.

## Abbreviations

GDM: Gestational Diabetes Mellitus; GWG: Gestational Weight Gain; HIV/AIDS: Human immunodeficiency virus/ Acquired immune deficiency syndrome; LMICs: Low and Middle Income Countries; MCH: Maternal and Child Health; NCD: Non-communicable Diseases; SES: Socioeconomic status; WHO: World Health Organization; UNDP: United Nations Development Program; UNESCO: United Nations Educational Scientific and Cultural Organization; UNICEF: United Nations Children's Fund.

## Competing interests

PDG and MAH have received honoraria and travel costs/expenses for speaking at conferences sponsored by nutrition companies (Nestle, Danone, Abbott). Since 2010, RCWM has received speaker honoraria at academic meetings supported by Astra Zeneca, Boehringer-Ingelheim, Danone, Eli Lilly, Nestle and Pfizer. All proceeds have been donated to the Chinese University of Hong Kong to support diabetes research.

## Authors’ contributions

All authors participated in deciding content, reviewing evidence, and writing this review. All authors read and approved the final manuscript.

## Pre-publication history

The pre-publication history for this paper can be accessed here:

http://www.biomedcentral.com/1471-2458/12/1025/prepub
